# Reduction in Exposure to Selected Harmful and Potentially Harmful Constituents Approaching Those Observed Upon Smoking Abstinence in Smokers Switching to the Menthol Tobacco Heating System 2.2 for 3 Months (Part 1)

**DOI:** 10.1093/ntr/ntz013

**Published:** 2019-02-05

**Authors:** Christelle Haziza, Guillaume de La Bourdonnaye, Andrea Donelli, Valerie Poux, Dimitra Skiada, Rolf Weitkunat, Gizelle Baker, Patrick Picavet, Frank Lüdicke

**Affiliations:** PMI R&D, Philip Morris Products S.A., Neuchâtel, Switzerland

## Abstract

**Introduction:**

The Tobacco Heating System (THS) is a “heat-not-burn” tobacco product designed to generate significantly lower levels of harmful and potentially harmful constituents (HPHCs) and present lower risk of harm than cigarettes. This study assessed the exposure reduction to selected HPHCs in smokers switching to menthol Tobacco Heating System (mTHS) 2.2 compared with smokers continuing smoking menthol cigarettes (mCCs) and smoking abstinence (SA) for 5 days in a confined setting, followed by an 86-day ambulatory period.

**Methods:**

A total of 160 healthy adult US smokers participated in this randomized, three-arm parallel group, controlled clinical study. Biomarkers of exposure to 16 HPHCs were measured in blood and 24-hour urine. Safety was monitored throughout the study. Information was also gathered on product evaluation, product use, subjective effects, and clinical risk markers (co-publication Part 2).

**Results:**

Nicotine uptake was comparable in both exposure groups (mTHS:mCC ratio of 96% on day 90). On day 5, biomarker of exposure levels to other HPHCs were reduced by 51%–96% in the mTHS group compared with the mCC group, and these reductions were sustained for most biomarkers of exposure over ambulatory period. After 90 days of use, the level of satisfaction with mTHS and suppression of urge to smoke were comparable to mCC.

**Conclusion:**

Switching from mCCs to mTHS significantly reduced the exposure to HPHCs to levels approaching those observed in subjects who abstained from smoking for the duration of the study.

**Implications:**

This study compared the impact of switching to mTHS on biomarkers of exposure, relative to continued smoking or SA.

**Clinical Significance:**

- Switching to mTHS 2.2 led to significant reductions of exposure (total NNAL, total NNN, COHb, MHBMA, 3-HPMA, S-PMA, Total 1-OHP, 4-ABP, 1-NA, 2-NA, o-tol, CEMA, HEMA, HMPMA, and B[a]P) after 5 days in confinement, which were maintained throughout the subsequent ambulatory period of 86 days. The reductions were similar to those observed upon SA.
- Moderate compliance in the mTHS group during the ambulatory period was indicative of dual use of mTHS and mCC.
- mTHS provides an acceptable alternative to mCC for adult smokers.

**Trial Registration:**

NCT01989156 (ClinicalTrials.gov).

## Introduction

Novel tobacco products, aimed at reducing the exposure to harmful and potentially harmful constituents (HPHCs) and, eventually, the risk of smoking-related diseases, are key to the harm reduction strategy adopted by Philip Morris International (PMI). PMI’s Tobacco Heating System (THS) is a candidate modified risk tobacco product defined by the US Family Smoking Prevention and Tobacco Control Act as “any tobacco product that is sold or distributed for use to reduce harm or the risk of tobacco related disease associated with commercially marketed tobacco products.”

The menthol Tobacco Heating System (mTHS), a heat-not-burn product created by PMI, has been developed to offer an alternative to menthol cigarette (mCC) smokers by replicating the ritual, taste, sensory characteristics, and nicotine delivery of mCC smoking.^1^ Also, heating tobacco rather than burning it generates a far less complex aerosol compared with cigarette smoke, resulting in a substantial reduction or elimination of, and thus exposure to, HPHCs.^2,3^

Previous clinical studies on heated tobacco products, focusing on maximizing internal validity by implementing strict control of product distribution and use, have demonstrated reduced exposure following *ad libitum* use over a short period of time (5–8 days).^4,5^ As this approach does not reflect real-world circumstances and actual use behavior over an extended period, PMI conducted a global assessment program for THS that included 3-month studies in Japan and the United States based on a 1-week confinement and a subsequent ambulatory period. In Japan, it was found that exposure reduction was largely maintained throughout the ambulatory period, and study participants mostly adhered to their randomized product allocation.^6^

In this study, US smokers were randomized to *ad libitum* use of mCC, mTHS, or smoking abstinence (SA). As in the previous study in Japan, the protocol included a 5-day confinement period to assess the exposure to nicotine and the maximum possible reductions of exposure to HPHCs in a well-controlled environment with strict control of product distribution, followed by an 86-day ambulatory period to assess real-life effects. Specifically, exposure to nicotine and to 16 HPHCs, including 14 HPHCs recommended for measurement by the US Food and Drug Administration (FDA), were assessed.^7^ Also, product use (including adherence to product allocation), nicotine dependence, subjective effects (including withdrawal symptoms and urge to smoke), product evaluation, mutagenicity, and enzymatic activity were assessed, and safety was monitored continuously.

## Methods

This study was performed in accordance with International Council for Harmonisation/Good Clinical Practice guidelines and the Declaration of Helsinki, 2008.^8,9^ The study was conducted in Dallas, Texas, and Daytona Beach, Florida, between December 2013 and October 2014, after approval by MidLands Independent Review Board in July 2013, and was published on ClinicalTrials.gov (NCT01989156).

### Design

The study was composed of four main periods. After the screening period, from day −30 to day −3, which included a product trial, subjects were enrolled (day −2) and randomized (day 0) in a 2:1:1 ratio to the mTHS, mCC, and SA groups. Randomization was stratified by sex and daily mCC consumption quotas (those smoking 10–19 mCCs and those smoking greater than 19 mCCs per day). In each arm, each sex and each of the smoking strata had a quota applied to ensure they represented at least 40% of the total randomized population. The 5-day confinement period (day 1 to day 5) was followed by an 86-day ambulatory period (day 6 to day 91) and an additional 28-day safety follow-up period in order to record spontaneously reported new adverse events (AEs) or serious adverse events (SAEs) and to monitor the active follow-up of ongoing AEs and SAEs by the site. On day −1 and day 0, all subjects smoked their own brand of mCC for baseline assessments (Supplementary Table 7). During the confinement period, subjects in the mTHS and mCC groups used exclusively *ad libitum* mTHS or their own brand of mCC, respectively, during the designated smoking hours (06:30 am–11:00 pm). Subjects in the SA group were asked to abstain from tobacco product use completely. On day 6, subjects were discharged from the study site and instructed to continue using their assigned product or to abstain from smoking for 86 days. Subjects were required to make three monthly visits of two consecutive days including one overnight stay each (day 30, 60, and 90) at the investigational site (Supplementary Figure 1).

### Participants

Healthy male and female US smokers, more than 22 years of age, were eligible. Subjects with safety-relevant diseases or with a history of alcohol and/or drug abuse, as well as pregnant or breastfeeding women, were excluded from the study. Subjects had to be within the body mass index range of 18.5–35 kg/m^2^ and had to meet all eligibility criteria listed in Supplementary Table 1 before being enrolled in the study.

### Products

The investigational product was mTHS 2.2. Maximum heating temperature is 350°C; per stick, menthol (2.62 mg/stick), nicotine (1.21 mg/stick), and glycerin (3.94 mg/stick) yields were obtained under the Health Canada Intense smoking regimen (Supplementary Table 2). Reference products were mCCs of the subjects’ preferred commercially available brands. Cigarettes were not provided to the subjects, who were asked to purchase their own preferred brand. Heatsticks, together with the THS 2.2 device, were provided to the subjects as THS 2.2 was not commercialized in the United States.

### Measurements

Biomarkers of exposure were assessed either in 24-hour urine or in blood. Measurements were taken daily from day −1 to day 5 and on the day 30, 60, and 90 visits. During the confinement period, the urine collection started post first void early in the morning on the study day and ended nearly 24 hours later with a last void. During the sampling period, all urine passed was collected. During the ambulatory visits (two consecutive days including one overnight stay), the urine collection started on the first day of the ambulatory visits, and ended 24 hours later on the second.

Creatinine (creat) was measured in 24-hour urine for adjustment of the concentration of all urinary biomarkers. Blood sampling was collected in the evening of each day during the confinement period and late in the morning on the first day of the ambulatory visit.

Primary endpoints included carboxyhemoglobin (COHb) measured as percent saturation of hemoglobin in blood, 3-hydroxypropylmercapturic acid (3-HPMA), monohydroxybutenyl mercapturic acid (MHBMA), and S-phenylmercapturic acid (S-PMA), measured as creatinine-adjusted urinary concentrations. Exposure to 4-(methylnitrosamino)-1-(3-pyridyl)-1-butanone (NNK) was assessed after 90 days of product use by measuring total 4-(methylnitrosamino)-1-(3-pyridyl)-1-butanol (total NNAL), a tobacco-specific biomarker of exposure with an elimination half-life of 10–18 days.^10^ The full list of biomarkers of exposure and the methods used to assess biomarkers, enzyme activity (CYP1A2), mutagenicity (Ames), and safety are detailed in the supplementary materials (Supplementary Methods and Measurements and Supplementary Table 3). As various studies have reported overlapping ranges in S-benzylmercapturic acid (S-BMA) levels, a biomarker of exposure to toluene, with only subtle differences observed between smokers and nonsmokers,^11–13^ and because excretion of S-BMA did not change across the three arms in this study and in other studies,^6,14^ S-BMA results are not reported here.

Compliance to the allocated product during the confinement period was ensured by strict dispensation of products, and the daily use per subject was recorded. During the ambulatory period, subjects were asked to record any use of mTHS, cigarettes (menthol or nonmenthol), nicotine replacement therapy, or nicotine/tobacco-containing products in an electronic diary. In addition, for the SA group, compliance was chemically verified using exhaled carbon monoxide (CO) breath tests (≤10 ppm). These tools permitted verification of compliance during the visits and between ambulatory visits.

Subjective effects of smoking were self-reported using the revised version of the Fagerström Test for Nicotine Dependence,^15^ the Minnesota Nicotine Withdrawal Scale ,^16^ the modified Cigarette Evaluation Questionnaire,^17^ and the brief version of the Questionnaire of Smoking Urges (QSU-brief).^18^

AEs, including abnormal laboratory findings, and SAEs were collected from the time of signature of the informed consent form until the end of the study. Safety assessment also included monitoring of respiratory symptoms (cough assessment by Visual Analogue Scale [VAS]), vital signs, physical examination, body weight, electrocardiogram (ECG), spirometry, and standard safety laboratory parameters (clinical chemistry, hematology, and urine analysis).

### Statistical Analysis

The sample size was calculated based on the expected mTHS:mCC biomarker of exposure concentration ratios, as observed in previous studies of heated tobacco products.^1,14^ A sample size of 160 participants, randomized 2:1:1 to the mTHS, mCC, and SA groups, respectively, was considered sufficient to attain 80% power to show reductions of at least 50% at day 5 for COHb, MHBMA, 3-HPMA, and S-PMA, and at day 90 for total NNAL, in the mTHS group compared with the mCC group, using one-sided tests with a 2.5% alpha level.

Safety was evaluated according to the actual product use in subjects who had at least one mTHS use, including the prerandomization product trial. Baseline characteristics and total NNAL were described for the full analysis set (ie, in subjects who had at least one postrandomization nonsafety assessment and either had at least one product use or were in the abstinence group).

The analysis of exposure effects was conducted in the per-protocol (PP) population,^19^ whose requirements were meeting all inclusion/exclusion criteria, correct randomization, correct sampling for the determination of the primary endpoints, and compliance with the randomized product allocation. PP compliance was determined separately for day 5 (from the period day 1 to day 5) and day 90 (from the period day 60 to day 90).

During the confinement period, strict compliance with the randomized product allocation was required for the PP, implying exclusive product use in the mTHS and mCC groups and complete smoking abstinence in the SA group based on the subject’s product use recorded in the log at site. In addition, for SA, abstinence from smoking was verified daily with a CO breath test of less than 10 ppm. From day 60 to day 90 of the ambulatory period, for subjects randomized to mTHS and SA groups, compliance to mTHS use or SA for the PP was defined as no more than two mCCs on a single day or more than 0.5 mCCs on average for the PP population based on the subject’s self-reported product use in the electronic diary. In addition, from day 60 to day 90 of the ambulatory period, full compliance was defined as exclusive compliance to the allocated product for subjects randomized to mTHS and cigarette arms and strict abstinence from smoking in the SA group based on self-reporting. A CO breath test of less than 10 ppm was required in the SA group.

General linear model estimates of mTHS:mCC ratios and 95% confidence intervals were calculated separately for log-transformed biomarker values adjusted for sex, average daily mCC consumption, and baseline biomarker level, for the day 5 and day 90 PP populations. All statistical analyses were performed with SAS v. 9.1.3.

## Results

### Participants

The safety population included 165 subjects who had tried mTHS, of which 160 (full analysis set) were randomized to mTHS (80), mCC (41), and SA (39). No major differences in sex, age, body mass index, daily cigarette consumption, and mean Fagerström Test for Nicotine Dependence total scores were observed among the subjects of the three study groups at baseline (Table 1).

**Table 1.** Baseline Characteristics by Randomization Group

Variables	mTHS	mCC	SA	Total
*N*	80	41	39	160
Age (y)				
Mean ± SD	39.2 ± 11.72	33.7 ± 10.17	38.8 ± 11.42	37.7 ± 11.45
Range	22–66	23–60	22–58	22–66
Sex, *n* (%)				
Male	48 (60.0)	24 (58.5)	24 (61.5)	96 (60.0)
Female	32 (40.0)	17 (41.5)	15 (38.5)	64 (40.0)
Race, *n* (%)				
White	49 (61.3)	28 (68.3)	22 (56.4)	99 (61.9)
Black or African American	23 (28.8)	11 (26.8)	17 (43.6)	51 (31.9)
Other	7 (8.8)	2 (4.9)	0	9 (5.6)
Missing	1 (1.3)	0	0	1 (0.6)
BMI (kg/m^2^)				
Mean ± SD	27.0 ± 4.11	25.8 ± 3.67	26.2 ± 3.76	26.5 ± 3.93
Range	19.1–34.9	18.6–32.9	19.4–34.3	18.6–34.9
FTND total score				
Mean ± SD	5.6 ± 2.25	5.5 ± 1.67	5.7 ± 2.14	5.6 ± 2.08
Range	0–10	1–9	2–9	0–10
Daily mCC consumption, *n* (%)				
10–19	43 (53.8)	21 (51.2)	18 (46.2)	82 (51.3)
>19	36 (45.0)	20 (48.8)	21 (53.8)	77 (48.1)
Missing	1 (1.3)	0	0	1 (0.6)

BMI = body mass index; FTND = Fagerström Test for Nicotine Dependence (revised version); mCC = menthol cigarette; mTHS = Tobacco Heating System 2.2 Menthol; SA = smoking abstinence.

For 13 subjects, an incorrect stratification factor was used for the randomization (mCC consumption at enrolment whereas mCC consumption was to be used). As for these subjects, the randomization procedure was incorrectly followed, these subjects were declared as misrandomized. This protocol deviation was considered major and leading to exclusion from the PP population.

On day 5, the PP criteria were fulfilled by 75, 35, and 24 subjects of the mTHS, mCC, and SA study groups, respectively. All of them were fully compliant with their allocated product exposure. Substantial discontinuation and nonabstinence (five subjects each) occurred in the SA group during confinement.

**Figure 1.**
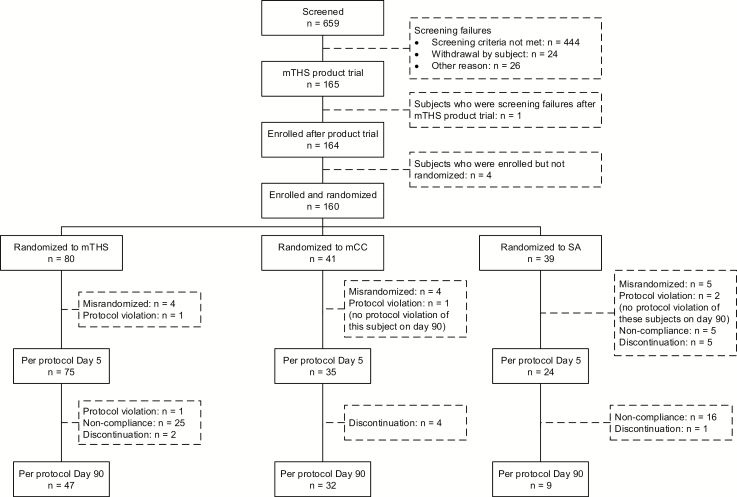
Disposition of subjects. mCC = menthol cigarette; mTHS = Tobacco Heating System 2.2 Menthol; SA = smoking abstinence.

On day 90, the PP criteria were fulfilled by 47, 32, and nine subjects of the mTHS, mCC, and SA study groups, respectively, and full compliance to the allocated product in the PP population was determined in 41, 31, and seven subjects in the mTHS, mCC, and SA study group, respectively. By day 90, 16 SA subjects of the day 5 PP population did not abstain from smoking. The detailed disposition of subjects is presented in Figure 1.

### Biomarkers of Exposure

**Figure 2.**
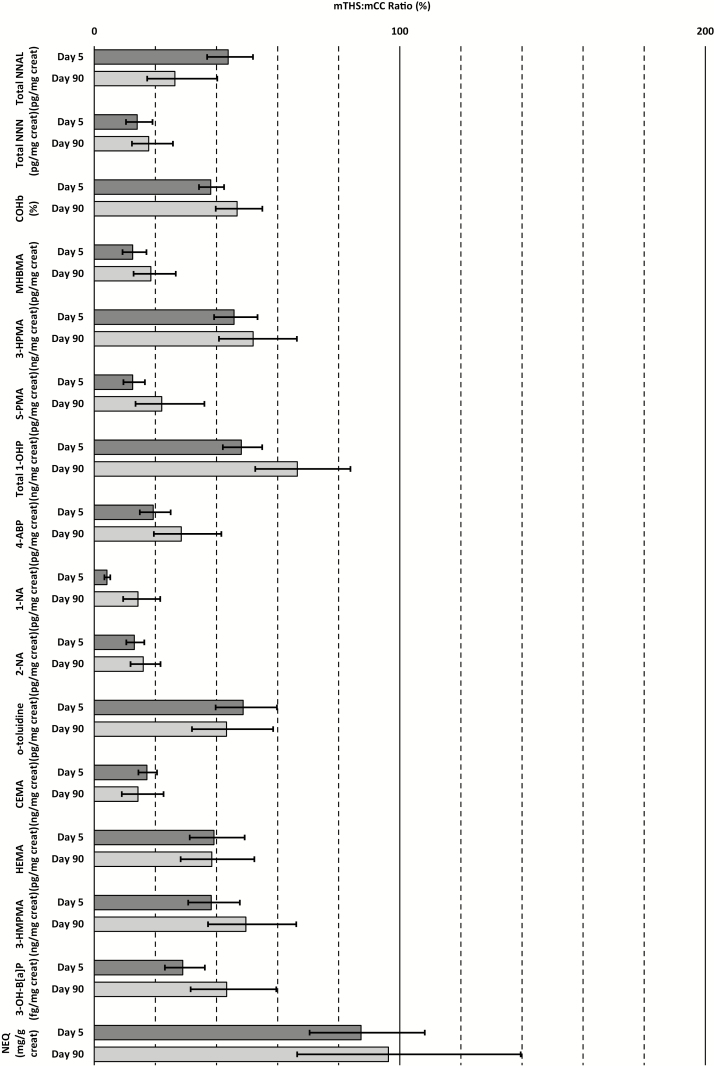
Biomarkers of exposure mTHS:mCC ratios (%) and 95% confidence intervals at day 5 (dark gray) and day 90 (light gray)—PP population. 4-ABP = 4-aminobiphenyl; CEMA = 2-cyanoethylmercapturic acid; COHb = carboxyhemoglobin; Creat = creatinine; 2-HEMA = 2-hydroxyethylmercapturic acid; 3-HMPMA = 3-hydroxy-1-methylpropylmercapturic acid; 3-HPMA = 3-hydroxypropylmercapturic acid; mCC = menthol cigarette; MHBMA = monohydroxybutenyl mercapturic acid; mTHS = Tobacco Heating System 2.2 Menthol; 1-NA = 1-aminonaphtalene; 2-NA = 2-aminonaphthalene; NEQ = nicotine equivalent; Total NNAL = total 4-(methylnitrosamino)-1-(3-pyridyl)-1-butanol; Total NNN = total *N*-nitrosonornicotine; 3-OH-B[a]P = 3-hydroxy-benzo[*a*]pyrene; Total 1-OHP = total 1-hydroxypyrene; o-tol = o-toluidine; PP = per-protocol; SA = smoking abstinence; S-PMA = S-phenylmercapturic acid.

Baseline levels of primary endpoints were comparable among the three groups, except for MHBMA concentrations, which were higher in the mTHS group than in the mCC and SA groups (Table 2). Average COHb, 3-HPMA, MHBMA, and S-PMA levels were reduced for mTHS, as compared with mCC, by 62%, 54%, 87%, and 87%, respectively, on day 5, and total NNAL level, assessed on day 90, was reduced by 74% (Figure 2).

**Table 2.** Geometric Mean (95% CI) of Primary (*) and Secondary Endpoints—PP Population

	*n*	mTHS	*n*	mCC	*n*	SA	Ratio mTHS:mCC
Total NNAL (pg/mg creat)^a,^*							
Day 5 analysis							
Baseline	67	150.01 (119.80 to 187.83)	30	147.90 (104.97 to 208.39)	21	142.18 (94.14 to 214.73)	
Day 5	73	57.04 (46.17 to 70.48)	34	120.29 (82.09 to 176.26)	22	54.74 (36.17 to 82.83)	43.81 (36.92 to 51.97)
Day 90 analysis							
Baseline	43	155.34 (116.54 to 207.05)	29	129.47 (94.72 to 176.95)	9	108.38 (57.04 to 205.94)	
Day 90	47	47.53 (34.80 to 64.91)	32	152.11 (108.38 to 213.47)	9	48.63 (17.69 to 133.71)	26.41 (17.31 to 40.26)
Total NNN (pg/mg creat)^b^							
Day 5 analysis							
Baseline	67	6.87 (5.48 to 8.62)	30	6.70 (4.78 to 9.38)	21	5.32 (3.42 to 8.28)	
Day 5	73	0.90 (0.71 to 1.13)	34	6.14 (4.42 to 8.53)	22	0.12 (0.09 to 0.15)	14.06 (10.38 to 19.06)
Day 90 analysis							
Baseline	43	7.80 (5.76 to 10.56)	29	5.66 (4.17 to 7.68)	9	4.48 (2.37 to 8.47)	
Day 90	47	0.94 (0.72 to 1.23)	32	4.47 (3.24 to 6.17)	9	0.26 (0.11 to 0.63)	17.80 (12.31 to 25.75)
COHb (%)—Evening value*							
Day 5 analysis							
Baseline	74	6.66 (6.23 to 7.13)	34	6.16 (5.59 to 6.80)	23	6.54 (5.79 to 7.38)	
Day 5	74	2.37 (2.23 to 2.51)	34	6.07 (5.52 to 6.68)	23	2.42 (2.08 to 2.81)	38.14 (34.24 to 42.47)
Day 90 analysis							
Baseline	47	6.49 (5.91 to 7.12)	32	6.23 (5.71 to 6.80)	9	5.79 (4.50 to 7.45)	
Day 90	47	2.66 (2.40 to 2.94)	32	5.62 (5.00 to 6.32)	9	2.84 (1.93 to 4.16)	46.76 (39.75 to 55.00)
MHBMA (pg/mg creat)*							
Day 5 analysis							
Baseline	65	1416.94 (1137.42 to 1765.14)	30	922.66 (596.93 to 1426.12)	21	1145.57 (721.09 to 1819.91)	
Day 5	71	113.01 (99.03 to 128.96)	33	760.36 (457.27 to 1264.34)	22	93.99 (75.54 to 116.94)	12.58 (9.27 to 17.05)
Day 90 analysis							
Baseline	43	1429.33 (1069.75 to 1909.77)	29	948.90 (616.80 to 1459.81)	9	595.28 (251.15 to 1410.96)	
Day 90	47	260.98 (205.28 to 331.79)	32	1040.71 (677.79 to 1597.94)	9	243.00 (128.12 to 460.90)	18.52 (12.85 to 26.67)
3-HPMA (ng/mg creat)*							
Day 5 analysis							
Baseline	67	721.15 (645.68 to 805.44)	30	777.25 (655.30 to 921.88)	21	711.68 (555.64 to 911.52)	
Day 5	73	283.88 (259.80 to 310.20)	34	655.19 (530.57 to 809.08)	22	151.43 (126.56 to 181.19)	45.77 (39.22 to 53.41)
Day 90 analysis							
Baseline	43	739.73 (643.81 to 849.94)	29	727.91 (609.42 to 869.44)	9	543.55 (344.91 to 856.58)	
Day 90	47	314.05 (281.51 to 350.34)	32	606.10 (468.27 to 784.48)	9	177.90 (90.83 to 348.41)	52.02 (40.80 to 66.33)
S-PMA (pg/mg creat)*							
Day 5 analysis							
Baseline	65	1510.14 (1213.34 to 1879.53)	30	1433.17 (1032.20 to 1989.89)	21	1488.78 (1012.87 to 2188.30)	
Day 5	71	133.64 (111.94 to 159.55)	33	1062.05 (685.84 to 1644.62)	22	133.11 (92.18 to 192.21)	12.58 (9.54 to 16.58)
Day 90 analysis							
Baseline	43	1479.21 (1114.94 to 1962.50)	29	1397.51 (1003.91 to 1945.42)	9	937.34 (429.73 to 2044.54)	
Day 90	47	314.02 (219.66 to 448.93)	32	1218.56 (822.54 to 1805.25)	9	181.62 (72.23 to 456.60)	22.08 (13.52 to 36.06)
Total 1-OHP (pg/mg creat)^c^							
Day 5 analysis							
Baseline	65	146.56 (127.08 to 169.03)	30	139.92 (111.20 to 176.05)	22	107.62 (85.31 to 135.76)	
Day 5	71	64.87 (57.55 to 73.12)	33	135.14 (111.12 to 164.35)	22	47.80 (35.48 to 64.40)	48.11 (42.11 to 54.96)
Day 90 analysis							
Baseline	43	160.27 (135.86 to 189.05)	29	129.15 (101.63 to 164.12)	9	90.25 (69.36 to 117.43)	
Day 90	47	117.77 (98.44 to 140.89)	32	163.80 (132.71 to 202.16)	9	70.19 (50.22 to 98.11)	66.46 (52.67 to 83.84)
4-ABP (pg/mg creat)							
Day 5 analysis							
Baseline	67	10.76 (9.01 to 12.86)	30	11.59 (9.42 to 14.26)	21	9.78 (7.14 to 13.38)	
Day 5	73	1.76 (1.50 to 2.08)	34	9.63 (7.76 to 11.95)	22	1.44 (1.06 to 1.97)	19.31 (14.90 to 25.01)
Day 90 analysis							
Baseline	43	11.46 (9.00 to 14.59)	29	10.61 (8.69 to 12.95)	9	7.71 (4.85 to 12.25)	
Day 90	47	3.77 (2.88 to 4.93)	32	11.31 (8.75 to 14.61)	9	2.98 (1.39 to 6.37)	28.48 (19.51 to 41.58)
1-NA (pg/mg creat)							
Day 5 analysis							
Baseline	67	61.36 (52.78 to 71.33)	30	62.93 (51.07 to 77.54)	21	56.16 (41.01 to 76.91)	
Day 5	73	2.51 (2.18 to 2.89)	34	63.05 (51.83 to 76.71)	22	2.17 (1.58 to 2.97)	4.15 (3.28 to 5.25)
Day 90 analysis							
Baseline	43	63.50 (51.63 to 78.11)	29	59.73 (48.99 to 72.82)	9	43.10 (24.49 to 75.84)	
Day 90	47	9.64 (7.31 to 12.72)	32	59.69 (47.34 to 75.24)	9	5.90 (2.27 to 15.30)	14.29 (9.47 to 21.56)
2-NA (pg/mg creat)							
Day 5 analysis							
Baseline	67	17.45 814.75 to 20.64)	30	18.65 (14.82 to 23.47)	21	15.97 (11.56 to 22.06)	
Day 5	73	2.10 (1.85 to 2.39)	34	16.28 (13.08 to 20.26)	22	1.98 (1.52 to 2.58)	13.12 (10.49 to 16.40)
Day 90 analysis							
Baseline	43	18.16 (14.49 to 22.76)	29	17.23 (13.74 to 21.61)	9	11.90 (6.63 to 21.36)	
Day 90	47	3.21 (2.57 to 4.00)	32	17.29 (13.62 to 21.95)	9	3.03 (1.80 to 5.11)	16.04 (11.87 to 21.67)
o-tol (pg/mg creat)							
Day 5 analysis							
Baseline	67	98.12 (84.75 to 113.59)	30	114.59 (92.45 to 142.04)	21	94.65 (73.46 to 121.95)	
Day 5	73	42.20 (37.79 to 47.13)	34	92.68 (77.80 to 110.39)	22	34.05 (26.55 to 43.68)	48.72 (39.70 to 59.79)
Day 90 analysis							
Baseline	43	98.98 (80.81 to 121.22)	29	109.34 (88.23 to 135.51)	9	77.58 (55.27 to 108.88)	
Day 90	47	47.53 (39.00 to 57.92)	32	107.39 (84.14 to 137.07)	9	37.30 (28.60 to 48.66)	43.29 (32.00 to 58.55)
CEMA (ng/mg creat)							
Day 5 analysis							
Baseline	67	90.62 (78.59 to 104.48)	30	99.50 (80.67 to 122.71)	21	91.97 (69.22 to 122.18)	
Day 5	73	14.39 (12.30 to 16.83)	34	88.40 (71.73 to 108.94)	22	13.40 (10.06 to 17.86)	17.23 (14.44 to 20.55)
Day 90 analysis							
Baseline	43	92.18 (76.45 to 111.15)	29	91.35 (73.85 to 112.99)	9	75.87 (41.99 to 137.06)	
Day 90	47	14.12 (10.16 to 19.61)	32	87.35 (66.11 to 115.41)	9	13.81 (4.70 to 40.57)	14.29 (9.01 to 22.67)
HEMA (pg/mg creat)							
Day 5 analysis							
Baseline	67	3702.58 (2998.48 to 4572.00)	30	3520.78 (2596.02 to 4774.95)	21	3289.73 (2300.10 to 4705.13)	
Day 5	73	1145.34 (951.15 to 1379.18)	34	2903.31 (2163.19 to 3896.66)	22	1075.28 (762.54 to 1516.30)	39.19 (31.22 to 49.20)
Day 90 analysis							
Baseline	43	3848.11 (2981.31 to 4966.93)	29	3307.99 (2458.13 to 4451.69)	9	2906.82 (1340.36 to 6303.95)	
Day 90	47	1481.32 (1193.81 to 1838.07)	32	3265.62 (2275.30 to 4686.97)	9	1565.88 (892.46 to 2747.43)	38.49 (28.28 to 52.38)
HMPMA (ng/mg creat)							
Day 5 analysis							
Baseline	67	297.82 (262.69 to 337.64)	30	312.55 (257.11 to 379.94)	21	304.27 (235.97 to 392.34)	
Day 5	73	95.35 (82.67 to 109.98)	34	270.99 (216.76 to 338.79)	22	84.11 (61.73 to 114.60)	38.26 (30.73 to 47.64)
Day 90 analysis							
Baseline	43	312.73 (263.30 to 371.45)	29	289.28 (238.77 to 350.47)	9	254.00 (151.96 to 424.54)	
Day 90	47	105.12 (86.68 to 127.47)	32	215.20 (167.61 to 276.30)	9	93.92 (49.42 to 178.49)	49.63 (37.25 to 66.13)
B[a]P (fg/mg creat)							
Day 5 analysis							
Baseline	67	135.23 (113.24 to 161.49)	30	132.19 (103.81 to 168.33)	21	86.69 (66.36 to 113.24)	
Day 5	73	33.44 (29.29 to 38.16)	34	107.09 (82.19 to 139.54)	22	18.34 (13.51 to 24.88)	28.94 (23.14 to 36.20)
Day 90 analysis							
Baseline	43	157.19 (128.31 to 192.58)	29	118.11 (92.03 to 151.57)	9	90.52 (62.65 to 130.77)	
Day 90	47	61.27 (48.60 to 77.25)	32	116.04 (88.25 to 152.58)	9	46.50 (34.32 to 63.01)	43.33 (31.52 to 59.57)
NEQ (mg/g creat)^d^							
Day 5 analysis							
Baseline	67	7.52 (6.59 to 8.58)	30	8.30 (7.03 to 9.80)	21	7.68 (6.12 to 9.64)	
Day 5	73	6.74 (5.76 to 7.89)	34	8.55 (7.18 to 10.18)	22	0.23 (0.14 to 0.39)	87.33 (70.49 to 108.18)
Day 90 analysis							
Baseline	43	7.61 (6.31 to 9.17)	29	7.97 (6.72 to 9.44)	9	6.00 (4.20 to 8.56)	
Day 90	47	6.52 (5.24 to 8.11)	32	7.40 (5.81 to 9.43)	9	0.82 (0.20 to 3.21)	96.30 (66.43 to 139.59)

Baseline for day 5 and day 90 analysis comprised all subjects included in the PP population at confinement and ambulatory on day 90, respectively.

4-ABP = 4-aminobiphenyl; CEMA = 2-cyanoethylmercapturic acid; COHb = carboxyhemoglobin; Creat = creatinine; 2-HEMA = 2-hydroxyethylmercapturic acid; 3-HMPMA = 3-hydroxy-1-methylpropylmercapturic acid; 3-HPMA = 3-hydroxypropylmercapturic acid; mCC = menthol cigarette; MHBMA = monohydroxybutenyl mercapturic acid; mTHS = Tobacco Heating System 2.2 Menthol; *n* = number of subjects with valid measurements; 1-NA = 1-aminonaphtalene; 2-NA = 2-aminonaphthalene; NEQ = nicotine equivalent; NNAL = 4-(methylnitrosamino)-1-(3-pyridyl)-1-butanol; NNN = *N*-nitrosonornicotine; 3-OH-B[a]P = 3-hydroxy-benzo[*a*]pyrene; 1-OHP = 1-hydroxypyrene; o-tol = o-toluidine; PP = per-protocol; SA = smoking abstinence; S-PMA = S-phenylmercapturic acid.

^a^Total NNAL was determined as the molar sum of 4-(methylnitrosamino)-1-(3-pyridy1)-1-butanol and its O-glucuronide conjugate.

^b^Total NNN was determined as the molar sum of free and conjugated NNN.

^c^1-OHP was determined as the molar sum of 1-hydroxypyrene and its glucuronide and sulfate conjugates.

^d^NEQ was determined as the molar sum of nicotine, cotinine, and trans-3′-hydroxycotinine plus their respective glucuronide conjugates.

In all other tobacco-related biomarkers of exposure, average reductions ranging from 51% (o-toluidine [o-tol]) to 96% (1-aminonaphtalene [1-NA]) were observed in the mTHS group compared with the mCC group on day 5 (Table 2). These reductions were mostly sustained after 90 days of ambulatory mTHS use (48%–86% reduction), the exceptions being 1-hydroxypyrene (1-OHP; reduction by 52% on day 5 and by 34% on day 90) and benzo[*a*]pyrene (B[a]P; reduction by 71% on day 5 and by 57% on day 90; Figure 2). Generally, average levels of biomarkers of exposure measured in subjects who switched to mTHS were similar to those measured in subjects who abstained from smoking (Table 2).

On day 90, total NNAL and total *N*-nitrosonornicotine (NNN) levels were, respectively, 74% and 82% lower in the mTHS group than in the mCC group. Nicotine-equivalent urinary concentrations remained similar in the mCC and mTHS groups on day 5 and day 90, when decreases of approximately 13% and 3%, respectively, were observed (Figure 2). Nicotine-equivalent levels were very low in the SA group but increased during the ambulatory period (from 0.23 mg/g creat on day 5 to 0.82 mg/g creat on day 90; Table 2).

### CYP1A2

No relevant baseline group differences were observed for CYP1A2 activity, ranging from 114% to 122%. On day 5, CYP1A2 activity in the mTHS and SA groups decreased by almost 33% and 35%, respectively, but increased by 4% in the mCC group (Supplementary Table 4). Compared with baseline, a decrease by 32% and 35% of the CYP1A2 activity was observed on day 90 for the mTHS and SA groups, respectively, compared with a decrease by 17% in the mCC group.

### Markers of Mutagenicity

Median levels of mutagenicity based on the Ames assay ranged from 17 384 (mCC group) to 25 823 revertants over 24 hours (rev/24 h; SA group) at baseline. Despite the high variability, there was a clear trend of decreasing urine mutagenicity values after 5 days of switching to mTHS (5801 rev/24 h) or SA (3920 rev/24 h), whereas an increase was observed in the mCC group (30 609 rev/24 h). On day 90, the mutagenicity levels were similar to those seen on day 5 in all groups (Supplementary Table 5).

### Subjective Effects

As of day 1 and until day 30, the product evaluation scores for all modified Cigarette Evaluation Questionnaire domains were lower in the mTHS group compared with the mCC group, except for aversion. From day 30 onward, all subscale scores were comparable between mTHS and mCC and remained stable afterwards at levels similar to the baseline (Supplementary Figure 2).

The average total QSU-brief urge to smoke scores were similar for all three groups at baseline (4.2, 4.2, and 4.0 for the mTHS, mCC, and SA groups, respectively) and remained stable and similar throughout the study for the mTHS and mCC groups, whereas the total score for the SA group increased from baseline to 5.2 (95% CI = 4.6 to 5.7) on day 1 and then decreased continuously to 4.0 (95% CI = 3.2 to 4.8) on day 5 (Supplementary Figure 3).

The Minnesota Nicotine Withdrawal Scale scores followed the same time course as those of the QSU-brief questionnaire (Supplementary Figure 4).

### Safety

Among the 165 subjects who were exposed to mTHS prior to randomization, two SAEs (diabetic ketoacidosis and sinusitis) were reported by one subject who was enrolled but not randomized. No SAE was reported in the postrandomization period.

Prior to randomization, there were 84 AEs reported in 62 subjects (37.6%), with the majority classified as mild. The rates of 195 mostly mild or moderate postrandomization AEs were comparable in the mTHS group (114 AEs in 52 of 80 subjects) and the SA group (49 AEs in 23 of 39 subjects) and slightly lower in the mCC group (32 AEs in 20 of 41 subjects). A summary of AEs experienced after randomization by more than one subject is provided in Supplementary Table 6. During the study, seven AEs in the mTHS and one AE in the mCC groups, respectively, were considered as being related to the tobacco products in use. They were all of mild intensity. The seven AEs reported in the THS group were linked to dry mouth, salivary hypersecretion, CO diffusing capacity decreased, sneezing, upper airway cough syndrome, noncardiac chest pain, and cough. The AE reported in the CC group was CO diffusing capacity decreased.

VAS-based assessment scores of cough during the confinement period decreased from 32.3 to 19.2 in the mTHS group and from 29.1 to 16.4 in the mCC group. On day 90, the average VAS score was lower in the mTHS group (21.8) compared with the mCC group (45.3). In the SA group, the VAS score increased from 13.9 on day 1 to 29.4 on day 6. In the ambulatory period, only one SA subject reported cough on day 30 (VAS score of 4.0), and no subject reported cough on days 60 and 90.

There were no clinically relevant abnormalities in vital signs or electrocardiograms, and no safety-relevant changes in lung function occurred during the study.

## Discussion

mTHS was developed to reduce or eliminate the formation of HPHCs in aerosol through heating and not burning tobacco while preserving, as much as possible, the taste, sensory experience, nicotine delivery profile, and ritual characteristics of mCC. This study in the United States with mTHS 2.2 demonstrated sustained exposure reduction to selected HPHCs in smokers switching to mTHS. The reductions were benchmarked against the lowest risk option for smokers: smoking abstention.

Significant and sustained reductions in the levels of biomarkers of exposure were observed following switching from mCC to mTHS throughout the entire exposure period. The reductions ranged from 51% to 96% and from 34% to 86% on day 5 and day 90, respectively, in subjects who switched to mTHS as compared with those who continued to smoke. The observed reductions in the mTHS group approached the levels observed in the SA group.

Baseline COHb levels were in agreement with levels in smokers as reported by the Institute of Medicine,^20^ with a range of 3.4%–7.1%, and remained stable throughout the study in the mCC group. By contrast, in the mTHS group the levels dropped to values similar to those in the SA group as of day 1. On day 90, the average levels were 2.7% and 2.8% in the mTHS and the SA groups, respectively, slightly exceeding what could be expected based on the available literature. The Agency for Toxic Substances and Disease Registry reports that COHb levels at least 2.4% may have adverse cardiovascular effects in subjects with compromised cardiovascular function^21^; similarly, the World Health Organization states that a COHb level of 2.5% should not be exceeded so as to prevent untoward hypoxic effects in the nonsmoking population with coronary artery diseases. The slightly higher levels observed are unlikely an effect of mTHS, as they also occurred in the SA group. Rather, incomplete compliance, and upward quantification bias at low concentrations^22^ appear to be the most likely explanation. Underestimation of measurements was reported across types of spectrophotometers (CO-oximeters) for values less than 2.5%. Environment factors could have also played a role.^23^

The confinement period served to ensure strict control of product allocation by preventing dual use and uncontrolled smoking. Owing to the high level of control during the confinement period, the internal validity of the findings is maximized. The relatively short confinement period, however, reduced the chance of detecting changes in biomarkers with longer half-lives, such as total NNAL. By continuing to monitor the biomarkers under ambulatory conditions, it was found that total NNAL concentration decreased further between day 5 and day 90. Considering the long half-life of total NNAL ranging between 10 and 45 days,^24,25^ the levels of total NNAL could be used to objectively estimate long-term abstinence from smoking in both mTHS and SA groups. At both day 5 and day 90, the levels of total NNAL were reduced from baseline, reaching absolute values similar between the mTHS and SA groups (57 and 55 pg/mg creat at days 5 and 48 and 49 pg/mg creat at day 90 in mTHS and CC groups, respectively). These values are in accordance with literature, where values of 32 pg/mg creat were reported in smokers following 6 weeks of smoking cessation,^26^ and thus indicate long-term abstinence from smoking. Compared with the concentrations reported for total NNAL in nonsmokers (1.19 pg/mg creat), the concentrations measured in both mTHS and SA groups are slightly higher^27^ in accordance with the occasional smoking that was allowed in the PP population.

There was a downward trend of urine mutagenicity values after switching to mTHS, comparable to what was observed in the SA group. As the Ames assay is indicative of exposure to mutagens, these findings provide additional evidence of reduced exposure when switching to mTHS. The reduction of urine mutagenicity as early as 5 days after switching to mTHS is in line with the published half-life of smoking-related urine mutagenicity (approximately seven to 23 hours).^28^ The high variability of the urine mutagenicity values is likely explained by (1) the relative high sensitivity of this assay to diet, as reported in the literature,^29^ (2) individual metabolic differences, and (3) variability of the cellular-based assay itself.^30^

The CYP1A2 enzymes are monooxygenases involved in the activation of carcinogenic heterocyclic and aromatic amines^31^ and also catalyze many of the reactions involved in the metabolism of low therapeutic index drugs and in the synthesis of cholesterol, steroids, and other lipids.^32^ CYP1A2 expression is induced to a large extent by polycyclic aromatic hydrocarbons, which are found in cigarette smoke.^33^ In this study, CYP1A2 activity in smokers who switched to mTHS was reduced to levels similar to those observed upon SA, aligning with what is reported in the literature.^34^ The reduction was sustained throughout the ambulatory period, likely linked to the overall reduction of exposure to polycyclic aromatic hydrocarbons.

Exposure reduction, as demonstrated consistently across a large array of biomarkers, is necessary but is not sufficient in itself to demonstrate risk reduction. Acceptance of and adaptation to the product to support complete switching without dual use of mCC are equally important. compliance with product allocation was moderate in this study. The slightly smaller biomarker reductions in the mTHS compared with the SA group during the ambulatory period have to be considered in the context of the PP criteria, not fully excluding any cigarette smoking. Also, exposure to environmental confounders, such as food and pollution, as well as other characteristics associated with the real-life conditions of the ambulatory period (including exposure to secondhand smoke) can play a role.

The initial change in taste, sensorial experience, ritual, and differences in the ISO tar and nicotine yield of mTHS compared with the subjects’ own preferred brand of mCC are likely reasons for the observed differences in overall satisfaction at the beginning of the exposure period. Obviously, switching from mCC to mTHS requires some adaptation over time. Although the urge to smoke and withdrawal appear to adapt within a week, product evaluation scores require between 1 week and 1 month to approach levels close to mCC. It is noteworthy that similar levels of acceptability can be achieved for both mTHS and mCC.

There were no postrandomization SAEs reported, and no subject was discontinued because of an AE. The majority of AEs were mild in severity, although 12 severe AEs were reported during the ambulatory phase, all of which were due to abnormal clinical laboratory findings and were unrelated to product use. There were no clinically relevant changes in safety laboratory parameters, vital signs, physical examination, electrocardiogram, spirometry findings, or assessment of cough.

There are several limitations of this study that warrant mentioning, including the potential for dual use in the mTHS group and the possibility to resume mCC smoking in the SA group during the ambulatory period. Indeed, compliance levels were lower than those observed previously in Japan in a study on mTHS with a similar design.^35^ In spite of the lower compliance in this study, the biomarker of exposure findings were largely comparable to those observed in Japan.

## Conclusions

This study provides evidence of sustained exposure reduction after switching from mCC to mTHS comparable to levels observed in those who abstain from smoking for the duration of the study. mTHS provides an acceptable alternative to smokers with regard to taste, ritual, sensorial experience, and nicotine delivery, and therefore can be a suitable substitute for mCC for adult smokers.

## Funding

Philip Morris International is the sole source of funding and sponsor of this project.

## Supplementary Material

ntz013_suppl_Supplementary_Figure_1

ntz013_suppl_Supplementary_Figure_2

ntz013_suppl_Supplementary_Figure_3

ntz013_suppl_Supplementary_Figure_4

ntz013_suppl_Supplementary_Material
