# Supplementary material for: Reduction in Exposure to Selected Harmful and Potentially Harmful Constituents Approaching Those Observed Upon Smoking Abstinence in Smokers Switching to the Menthol Tobacco Heating System 2.2 for 3 Months (Part 1)
Source: Nicotine Tob Res. 2019 Feb 5;22(4):539–48. doi: 10.1093/ntr/ntz013 (PMC7164581; doi:10.1093/ntr/ntz013)

Supplementary Figure 1. Study Design.
Abbreviations: mCC = menthol cigarettes; mTHS = Tobacco Heating System 2.2 Menthol.

a The screening visit was scheduled one to four weeks before enrolment and included trial use of the mTHS.
b The participant continued using their preferred mCCs.
c Ad libitum use of mTHS or mCC. The products were dispensed to the participant according to the product allocation.
d Ad libitum use of mTHS or mCC according to the product allocation. Dual use of mTHS and mCC was possible in the mTHS group but not in the mCC group.
e The participants were asked to abstain from smoking and were provided with psychological support during the period of abstinence. The use of mTHS was strictly forbidden for participants in the SA group for the whole study duration.

Subjects were not discontinued from the study for the use of nicotine/tobacco-containing products other than their assigned product.

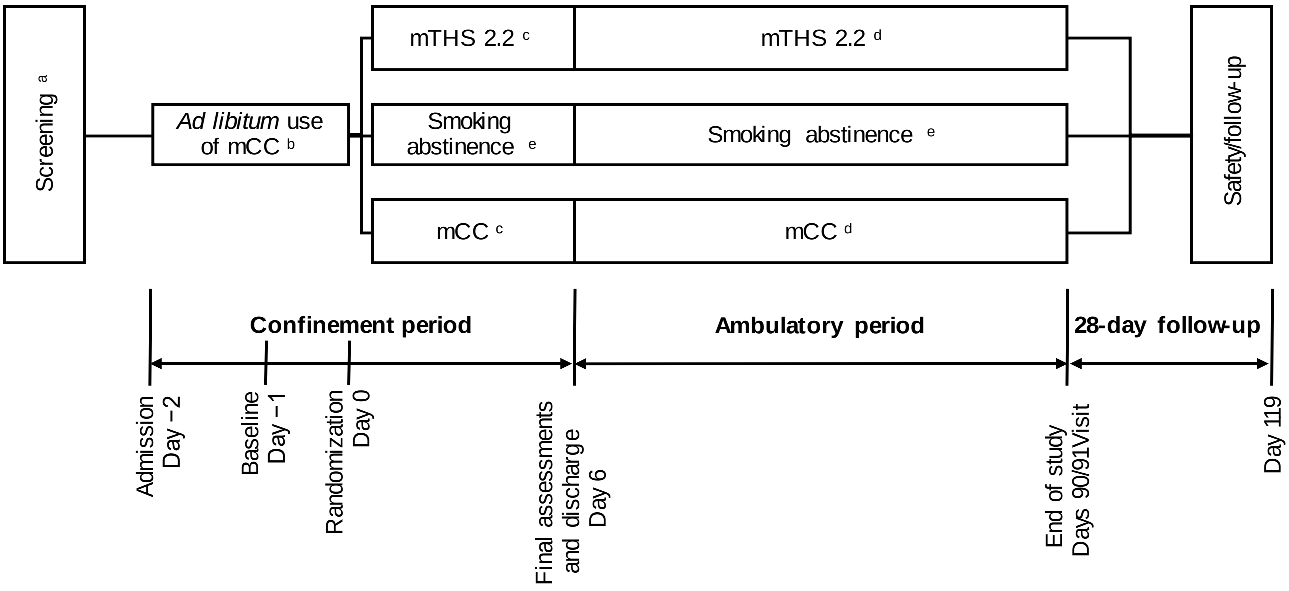

Supplement: ntz013_suppl_Supplementary_Figure_1 [file ntz013_suppl_supplementary_figure_1.pdf]
