# Supplementary material for: Reduction in Exposure to Selected Harmful and Potentially Harmful Constituents Approaching Those Observed Upon Smoking Abstinence in Smokers Switching to the Menthol Tobacco Heating System 2.2 for 3 Months (Part 1)
Source: Nicotine Tob Res. 2019 Feb 5;22(4):539–48. doi: 10.1093/ntr/ntz013 (PMC7164581; doi:10.1093/ntr/ntz013)

Supplementary Figure 3. QSU-Brief Version Total Scores (Means and 95% CIs) – PP Population.

Abbreviations: CI = confidence interval; mCC = menthol cigarette; mTHS = Tobacco Heating System 2.2 Menthol; SA = smoking abstinence.

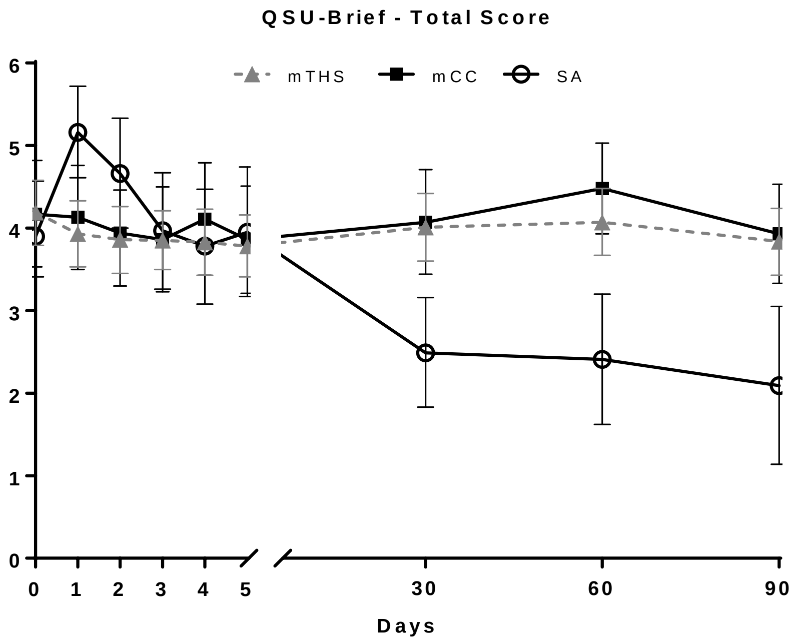

Supplement: ntz013_suppl_Supplementary_Figure_3 [file ntz013_suppl_supplementary_figure_3.pdf]
