# Supplementary material for: Reduction in Exposure to Selected Harmful and Potentially Harmful Constituents Approaching Those Observed Upon Smoking Abstinence in Smokers Switching to the Menthol Tobacco Heating System 2.2 for 3 Months (Part 1)
Source: Nicotine Tob Res. 2019 Feb 5;22(4):539–48. doi: 10.1093/ntr/ntz013 (PMC7164581; doi:10.1093/ntr/ntz013)
Supplement: ntz013_suppl_Supplementary_Material [file ntz013_suppl_supplementary_material.doc]

# SUPPLEMENTARY MATERIALS

## Methods and Measurements

Biomarkers of exposure to HPHCs were selected based on the HPHCs recommended for lowering in cigarette smoke, as defined by WHO [^33^](#_ENREF_33) and being part of the FDA draft guidance [^34^](#_ENREF_34), the HPHC being specific to the source of exposure with other sources being minor or non-existent, reflecting a specific toxic exposure (or being a reliable surrogate of exposure), and the biomarker of exposure exhibiting an apparent elimination half-life appropriate for the periods defined in this study. The list of HPHCs includes a broad variety of chemical classes and organ toxicity classes, as defined by the FDA (carcinogen, cardiovascular toxicant, respiratory toxicant, reproductive and development toxicant, addiction potential) [^9^](#_ENREF_9) .

The biomarker of exposure assays were validated as “fit-to-purpose” to meet the applicable portions of the FDA draft guidance. O-toluidine, 1-NA, 2-NA, 4-ABP, S-PMA, 1-OHP, B[a]P, Total NNAL, and Total NNN were measured in urine after hydrolysis. An acidic hydrolysis was employed for the measurement of the aromatic amines and S-PMA. 1-OHP, 3- B[a]P, Total NNAL, and Total NNN were hydrolysed enzymatically.

A direct analysis of urinary concentrations of MHBMA, 3-HPMA, HMPMA, CEMA, S-BMA, 8-epi-PGF2α-III, and 11-DTX-B2 was performed as follows. Clinical sample aliquots, supplemented with stable label internal standards, were extracted either by a validated liquid-liquid or solid-phase extraction approach. The extracts were injected onto a qualified liquid chromatography tandem mass spectrometry (LC-MS/MS) instrument and detected in multiple reaction monitoring mode.

Nicotine, cotinine, trans-3´-hydroxycotinine, caffeine (CAF), and paraxanthine (PX) were measured in plasma using validated assays to meet the FDA guidance on Bioanalytical Method Validation (2001). Clinical samples were assayed with the addition of stable label internal standards to the aliquoted sample. Both assays employed a solid phase extraction to concentrate and purify the target analytes. The extracts were injected onto a qualified LC-MS/MS instrument with positive ions detected in multiple reaction monitoring mode.

In addition to the total assays, nicotine equivalents were also measured with the direct analysis of nicotine, cotinine, trans-3´-hydroxycotinine, nicotine-N-glucuronide, cotinine-N-glucuronide, and trans-3´-hydroxycotinine-O-glucuronide in urine.

Urinary creatinine and COHb in whole blood were measured spectrophotometrically using CAP/CLIA validated assays.

Cytochrome P450 (CYP) 1A2 enzymatic activity was measured on Day 0, Day 5, and Day 90 and was based on the post-dose PX and CAF plasma molar concentrations approximately six hours (±15 minutes) after the intake of one Tomerumin® (LionCorp.) caffeine tablet (around 170mg caffeine) with 150 ml ±10 ml water [^31^](#_ENREF_31). CYP1A2 activity was assessed by measuring PX and CAF concentrations and calculating the PX/CAF molar metabolic ratio [^31^](#_ENREF_31).

Mutagenicity potential of urine of subjects, an indicator of exposure to mutagenic compounds, was evaluated by Labstat International ULC (Ontario, Canada) using the reverse mutation assay (Ames assay) tested on the Salmonella typhimurium strain YG1024 containing the plasmid pYG219 [^35^](#_ENREF_35), which encodes for the enzyme acetyl-coA: N-hydroxyarylamine O-acetyltransferase. This bacterial strain is sensitive towards mutagenicity of aromatic amines as well as nitro-containing chemicals. A zero control and four doses of urine concentrates were prepared from 24-hour urine collected on Day 0, Day 5, and Day 90 (in three replicates) and were incubated in the presence of S9 metabolic activation and overlayed on an Agar plate for 42 to 72 hours at 37°C. Then, the number of revertant colonies per plate was counted and expressed as number of revertant/mL. To evaluate urine mutagenicity, the activity per day expressed as rev/24h urine, was calculated by determining the slope of the dose-response function (revertants/ml of urine) on the basis of the number of revertant colonies and by multiplying the slope by the 24-hour urine volume of each subject. If the slope was negative, the activity indicator value was 0.

Safety evaluations included vital signs, physical examinations, body weight, electrocardiography, safety laboratory tests (clinical chemistry, hematology, and urinalysis), and AEs/SAEs. The intensity of AEs (graded as mild, moderate, or severe) and their relationship to the investigational products were also assessed. The considered SAEs included, but were not limited to, medical events that resulted in death, were life-threatening, required inpatient hospitalization or prolongation of existing hospitalization, resulted in persistent or significant disability/incapacity, or resulted from a congenital anomaly/birth defect.

Supplementary Table 1. Eligibility Criteria.

| **Inclusion Criteria** |
| --- |
| - Subject had signed the ICF and was able to understand the Subject Information Sheet and ICF. |
| - Subject was at a minimum 22 years of age. |
| - Smoking, apparently healthy subject as judged by the Principal Investigator based on all available assessments from the Screening period/Day of Admission (e.g., safety laboratory, spirometry, vital signs, physical examination, ECG, chest X-ray, and medical history). |
| - Subject smoked at least 10 commercially available mCCs per day (no brand restrictions), for the last four weeks (self-reporting). Furthermore, the subject had been smoking for at least the last three consecutive years. The smoking status was verified based on a urinary cotinine test (cotinine ≥ 200 ng/mL). |
| - The subject did not plan to quit smoking within the next six months. |
| - The subject was ready to comply with study protocol and to use THS 2.2 Menthol^*^.   *Readiness to use THS 2.2 Menthol was asked at Day of Admission after the product test. |
| **Exclusion Criteria** |
| - As per Principal Investigator’s judgment, the subject could not participate in the study for any reason (e.g., medical, psychiatric, and/or social reason). |
| - A subject who was legally incompetent or physically or mentally incapable of giving consent (e.g., emergency situation, under guardianship, subject in a social or sanitary establishment, prisoners, or subjects who are incarcerated involuntarily). |
| - As per Principal Investigator judgment, the subject had medical conditions that required or would require in the course of the study, medications or a medical intervention (e.g., start of treatment, surgery, hospitalization) that may have interfered with the study participation and/or study results or would have jeopardised the safety of the subject. |
| - Subject who had FEV_1_/FVC < 0.7 and FEV_1_ < 80% predicted value at post bronchodilator spirometry. |
| - Subject with asthma condition (FEV_1_/FVC < 0.75 and reversibility in FEV_1_ > 12% [or > 200 mL] from pre- to post-bronchodilator values). |
| - Subjects with renal insufficiency, as defined by serum creatinine levels of > 1.3 mg/dL for females and > 1.5 mg/dL for males. |
| - The subject had a BMI < 18.5 or ≥ 35 kg/m^2^. |
| - Any subject with a history of AEs linked to caffeine or caffeine-containing drugs (e.g., Vivarin), such as but not limited to hypersensitivity or allergy. |
| - The subject had used nicotine-containing products other than commercially available mCC (either tobacco-based products or NRT), as well as electronic cigarettes and similar devices, within four weeks prior to assessment. |
| - The subject had received medication (prescription or over-the-counter) within 14 days or within five half-lives of the drug (whichever is longer) prior to the Admission Day (Day -2) that had an impact on CYP1A2 or CYP2A6 activity. |
| - If a subject had received any medication (prescribed or over-the-counter) within 14 days prior to Screening or prior to the Admission Day (Day -2), it was to be decided at the discretion of the Principal Investigator if these could potentially interfere with the study objectives or subject’s safety. |
| - Concomitant use of NSAIDs or acetylsalicylic acid. |
| - The subject had a positive alcohol test and/or the subject had a history of alcohol abuse that could interfere with the subject’s participation in the study. - The subject had a positive urine drug test. |
| - Positive serology test for human immunodeficiency virus 1/2, hepatitis B, or hepatitis C. |
| - Donation or receipt of whole blood or blood products within three months prior to Admission. |
| - The subject or a first-degree relative (parent, sibling, child) was a current or former employee of the tobacco industry. |
| - The subject or a first-degree relative (parent, sibling, child) was an employee of the investigational site or any other parties involved in the study. |
| - The subject had participated in a clinical study within three months prior to the Screening Visit. |
| Additionally, women were excluded if:   - Subject was pregnant (did not have negative pregnancy tests at Screening and at Admission) or was breast feeding. - Subject did not agree to use an acceptable method of effective contraception.^*^   *Intrauterine device, intrauterine system, established use of oral/injectable/implantable/transdermal hormonal methods, barrier methods of contraception (condoms, occlusive caps) with spermicidal foam/gel/film/suppository, vasectomised partner(s), or true abstinence (periodic abstinence and withdrawal are not effective methods) from Screening until the end of the safety follow-up period. Hysterectomy, tubal ligation, bilateral oophorectomy, or post-menopausal status were reasons for not needing to use birth control. Post-menopausal status was defined as women who had not experienced menses for greater than 12 months. If a woman claimed she was post-menopausal but had had her menses within 12 months, a follicle-stimulating hormone test was to be performed and must have been within acceptable limits. |

Abbreviations: CYP1A2 = cytochrome P450 1A2; CYP2A6 = cytochrome P450 2A6; FEV_1_ = forced expiratory volume in 1 second; FVC = forced vital capacity; ICF = informed consent form; ISO = International Organization for Standardization; mCC = menthol cigarettes; mTHS = Tobacco Heating System 2.2 Menthol; NSAID = nonsteroidal anti-inflammatory drug.

The use of nicotine-containing products other than their own brand of mCCs as well as the use of medication with an impact on CYP2A6 activity, NSAIDs, or acetylsalicylic acid was not allowed. All participants were informed that they were free to discontinue from the study at any time. Subjects enrolled, but wanting to quit smoking, were encouraged to do so and were discontinued from the study before being referred to a smoking cessation counsellor.

Supplementary Table 2. HPHC Yields from mTHS Obtained Under HCI Machine-Smoking Conditions and Expressed on a Per Tobacco Stick Basis.

| Parameter | Unit | N | Mean ± SD |
| --- | --- | --- | --- |
| TPM | mg/stick | 4 | 43.54 ± 0.92 |
| Water | mg/stick | 4 | 29.73 ± 2.26 |
| Nicotine | mg/stick | 4 | 1.21 ± 0.06 |
| Carbon monoxide | mg/stick | 4 | 0.59 ± 0.07 |
| Menthol | mg/stick | 4 | 2.62 ± 0.07 |
| Glycerin | mg/stick | 4 | 3.94 ± 0.55 |

Abbreviations: HCI = Health Canada Intense; HPHC = harmful and potentially harmful constituent; mTHS = Tobacco Heating System 2.2 Menthol; N = number of determinations; SD = standard deviation, TPM = total particulate matter.

Supplementary Table 3. List of Primary (^*^) and Secondary Endpoints.

| **Biomarkers of Exposure** | **HPHC** | **Matrix** | **Laboratory** |
| --- | --- | --- | --- |
| ***Tobacco Specific*** |  |  |  |
| Total 4-(methylnitrosamino)-1-(3-pyridyl)-1-butanol (Total NNAL ^a*^) | 4-(methylnitrosamino)-1-(3-pyridyl)-1-butanone (NNK) | Urine | Celerion USA ^d^ |
| Total N-nitrosonornicotine (Total NNN) | N-nitrosonornicotine (NNN) | Urine | Celerion USA ^d^ |
| ***Tobacco Related*** |  |  |  |
| Monohydroxybutenyl mercapturic acid (MHBMA ^*^) | 1,3‑butadiene | Urine | Celerion CH ^e^ |
| 3-hydroxypropylmercapturic acid (3-HPMA ^*^) | Acrolein | Urine | Celerion USA ^d^ |
| S-phenylmercapturic acid (S-PMA ^*^) | Benzene | Urine | Celerion CH ^e^ |
| Carboxyhemoglobin (COHb ^*^) | Carbon monoxide | Blood | Celerion USA ^d^ |
| Total 1-hydroxypyrene (1-OHP ^b^) | Pyrene | Urine | Celerion USA ^d^ |
| 4-aminobiphenyl (4-ABP) | 4-aminobiphenyl (4-ABP) | Urine | Celerion USA ^d^ |
| 1-aminonaphthalene (1-NA) | 1-aminonaphthalene (1-NA) | Urine | Celerion USA ^d^ |
| 2-aminonaphthalene (2-NA) | 2-aminonaphthalene (2-NA) | Urine | Celerion USA ^d^ |
| o-toluidine (o-tol) | o-toluidine (o-tol) | Urine | Celerion USA ^d^ |
| 2-cyanoethylmercapturic acid (CEMA) | Acrylonitrile | Urine | Celerion USA ^d^ |
| 2-hydroxyethyl mercapturic acid (HEMA) | Ethylene oxide | Urine | Celerion USA ^d^ |
| 3-hydroxy-1-methylpropylmercapturic acid (3‑HMPMA) | Crotonaldehyde | Urine | Celerion USA ^d^ |
| 3-hydroxybenzo(a)pyrene (3-OH-B[a]P) | Benzo(a)pyrene (B[a]P) | Urine | Celerion USA ^d^ |
| S-benzylmercapturic acid (S-BMA) | Toluene | Urine | Celerion CH ^e^ |
| ***Nicotine Exposure*** |  |  |  |
| Nicotine | Nicotine | Plasma | Celerion USA ^d^ |
| Cotinine |  | Plasma | Celerion USA ^d^ |
| Nicotine equivalents (Neq ^c^): free nicotine, nicotine-glucuronide, free cotinine, cotinine-glucuronide, free trans-3’-hydroxycotinine, trans-3’-hydroxycotinine-glucuronide | Nicotine | Urine | Celerion USA ^d^ |

All biomarkers of exposure except COHb and creatinine were analysed by LC-MS/MS by independent contract laboratories. COHb and creatinine were measured by spectrophotometry, and carbon monoxide in exhaled breath was measured using the Micro+TM Smokerlyzer® device.

Biomarkers of exposure were measured on Day -1 to Day 5 and at Day 30, Day 60, and Day 90. Data obtained on Day -1 (baseline), Day 5, and Day 90 are reported.

^*^ Primary biomarker of exposure.

1. Total NNAL was determined as the molar sum of 4-(methylnitrosamino)-1-(3-pyridy1)-1-butanol and its O-glucuronide conjugate.
2. 1-OHP was determined as the molar sum of 1-hydroxypyrene and its glucuronide and sulfate conjugates.
3. NEQ was determined as the molar sum of nicotine, cotinine, and trans-3’-hydroxycotinine plus their respective glucuronide conjugates.
4. Celerion USA, Lincoln, NE, USA
5. Celerion Switzerland, Fehraltorf, Switzerland

Abbreviations: HPHC = harmful or potentially harmful smoke constituent.

Supplementary Table 4. CYP1A2 Activity at Baseline, Day 5, and Day 90 – PP Population.

|  |  | **n** | **mTHS** | **n** | **mCC** | **n** | **SA** | **Ratio mTHS:mCC * (95% CI)** |
| --- | --- | --- | --- | --- | --- | --- | --- | --- |
| *Day 5 Analysis* | |  |  |  |  |  |  |  |
| Baseline | Geometric Mean  (95% CI) | 74 | 117.6  (109.2; 126.7) | 34 | 121.6  (109.1; 135.5) | 23 | 114.0  (96.6; 134.5) |  |
| Day 5 | Geometric Mean  (95% CI) | 74 | 76.7  (70.3; 83.7) | 35 | 123.6  (112.6; 135.6) | 23 | 73.4  (60.3; 89.5) | 63.5 (58.3; 69.2) |
| Δ | % change from Baseline  (95% CI) | 74 | -32.8  (-36.8; -28.9) | 34 | 3.6  (-1.2; 8.3) | 23 | -34.5  (-39.7; -29.4) |  |
| *Day 90 Analysis* | |  |  |  |  |  |  |  |
| Baseline | Geometric Mean  (95% CI) | 47 | 114.2  (105.2; 124.0) | 31 | 118.5  (106.7; 131.6) | 9 | 140.5  (114.5; 172.3) |  |
| Day 90 | Geometric Mean  (95% CI) | 47 | 71.8  (63.1; 81.7) | 32 | 95.1  (81.9; 110.6) | 9 | 85.0  (54.4; 132.8) | 78.6 (66.4; 93.1) |
| Δ | % change from Baseline  (95% CI) | 47 | -32.0  (-40.5; -23.5) | 31 | -16.7  (-23.9; -9.5) | 9 | -35.4  (-53.5; -17.2) |  |

Abbreviations: CI = confidence interval; mCC = menthol cigarette; mTHS = Tobacco Heating System 2.2 Menthol; n = number of subjects with valid measurements; SA = smoking abstinence.

*Geometric least square mean ratio

Supplementary Table 5. Ames Testing at Baseline, Day 5, and Day 90; Expressed as Revertants Over 24-Hour Urine – PP Population.

|  |  | **mTHS** | **mCC** | **SA** |
| --- | --- | --- | --- | --- |
| *Day 5 Analysis* | |  |  |  |
| Baseline | n | 63 | 29 | 21 |
|  | M ± SD | 30251.2 ± 29094.3 | 24798.7 ± 19446.8 | 28259.4 ± 18515.3 |
|  | Median (Range) | 19463.2 (2416.2; 148367.2) | 17384.4 (0.0; 81244.2) | 25823.0 (5611.2; 80368.2) |
| Day 5 | n | 73 | 34 | 23 |
|  | M ± SD | 8438.3 ± 9523.2 | 36544.3 ± 29684.1 | 10806.3 ± 17858.4 |
|  | Median (Range) | 5800.7 (0.0; 43350.4) | 30608.7 (0.0; 129114.4) | 3920.2 (0.0; 80368.2) |
| *Day 90 Analysis* | |  |  |  |
| Baseline | n | 41 | 28 | 8 |
|  | M ± SD | 28352.9 ± 29045.7 | 23283.6 ± 17577.5 | 22469.7 ± 10997.9 |
|  | Median (Range) | 18668.8 (2416.2; 148367.2) | 17131.6 (0.0; 81244.2) | 24980.4 (6900.5; 35294.0) |
| Day 90 | n | 47 | 32 | 9 |
|  | M ± SD | 10308.5 ± 8809.0 | 28405.3 ± 21826.0 | 6322.9 ± 4817.1 |
|  | Median (Range) | 7439.1 (0.0; 53250.4) | 22047.5 (5968.1; 87139.8) | 6178.1 (0.0; 13607.2) |

Abbreviations: M = arithmetic mean; mCC = menthol cigarette; mTHS = Tobacco Heating System 2.2 Menthol; n = number of subjects with valid measurements; SA = smoking abstinence; SD = standard deviation.

Supplementary Table 6. Adverse Events [N, (%)] in More Than One Participant Following Randomization (by System Organ Class and Preferred Term).

| **System Organ Class**  **Preferred Term** | **mTHS** | | **mCC** | | **SA** | | **Overall** | |  |
| --- | --- | --- | --- | --- | --- | --- | --- | --- | --- |
| Subjects with any AE | 52 (65.0) | | 20 (48.8) | | 23 (59.0) | | 95 (59.4) | |  |
| Gastrointestinal disorders | 15 (18.8) | | 2 (4.9) | | 3 (7.7) | | 20 (12.5) | |  |
| Abdominal pain | 1 (1.3) | | 1 (2.4) | | 0 | | 2 (1.3) | |  |
| Constipation | 3 (3.8) | | 0 | | 0 | | 3 (1.9) | |  |
| Dry mouth | 2 (2.5) | | 0 | | 1 (2.6) | | 3 (1.9) | |  |
| Lip dry | 1 (1.3) | | 1 (2.4) | | 0 | | 2 (1.3) | |  |
| Nausea | 3 (3.8) | | 0 | | 1 (2.6) | | 4 (2.5) | |  |
| Toothache | 2 (2.5) | | 0 | | 1 (2.6) | | 3 (1.9) | |  |
| Infections and infestations | 5 (6.3) | | 5 (12.2) | | 1 (2.6) | | 11 (6.9) | |  |
| Upper respiratory tract infection | 3 (3.8) | | 4 (9.8) | | 1 (2.6) | | 8 (5.0) | |  |
| Injury, poisoning, and procedural complications | 5 (6.3) | | 4 (9.8) | | 3 (7.7) | | 12 (7.5) | |  |
| Administration related reaction | 0 | | 2 (4.9) | | 0 | | 2 (1.3) | |  |
| Ligament sprain | 3 (3.8) | | 0 | | 0 | | 3 (1.9) | |  |
| Muscle strain | 0 | | 0 | | 2 (5.1) | | 2 (1.3) | |  |
| Investigations | 27 (33.8) | | 11 (26.8) | | 12 (30.8) | | 50 (31.3) | |  |
| Aspartate aminotransferase increased | 1 (1.3) | | 1 (2.4) | | 0 | | 2 (1.3) | |  |
| Blood bilirubin increased | 1 (1.3) | | 0 | | 1 (2.6) | | 2 (1.3) | |  |
| Blood potassium increased | 2 (2.5) | | 1 (2.4) | | 0 | | 3 (1.9) | |  |
| Blood triglycerides increased | 2 (2.5) | | 2 (4.9) | | 2 (5.1) | | 6 (3.8) | |  |
| Forced expiratory volume decreased | 2 (2.5) | | 0 | | 0 | | 2 (1.3) | |  |
| Haemoglobin decreased | 11 (13.8) | | 4 (9.8) | | 6 (15.4) | | 21 (13.1) | |  |
| Lymphocyte count increased | 6 (7.5) | | 2 (4.9) | | 1 (2.6) | | 9 (5.6) | |  |
| Neutrophil count decreased | 4 (5.0) | | 0 | | 0 | | 4 (2.5) | |  |
| Vital capacity decreased | 2 (2.5) | | 0 | | 0 | | 2 (1.3) | |  |
| Carbon monoxide diffusing capacity decreased | 1 (1.3) | | 1 (2.4) | | 1 (2.6) | | 3 (1.9) | |  |
| Metabolism and nutrition disorders | 2 (2.5) | | 0 | | 2 (5.1) | | 4 (2.5) | |  |
| Hypertriglyceridaemia | 2 (2.5) | | 0 | | 0 | | 2 (1.3) | |  |
| Musculoskeletal and connective tissue disorders | 2 (2.5) | | 0 | | 1 (2.6) | | 3 (1.9) | |  |
| Back pain | 1 (1.3) | | 0 | | 1 (2.6) | | 2 (1.3) | |  |
| Nervous system disorders | 7 (8.8) | | 1 (2.4) | | 4 (10.3) | | 12 (7.5) | |  |
| Dizziness | 1 (1.3) | | 0 | | 2 (5.1) | | 3 (1.9) | |  |
| Headache | 4 (5.0) | | 1 (2.4) | | 3 (7.7) | | 8 (5.0) | |  |
| Renal and urinary disorders | 1 (1.3) | | 0 | | 3 (7.7) | | 4 (2.5) | |  |
| Glycosuria | 1 (1.3) | | 0 | | 1 (2.6) | | 2 (1.3) | |  |
| Respiratory, thoracic, and mediastinal disorders | 9 (11.3) | | 4 (9.8) | | 3 (7.7) | | 16 (10.0) | |  |
| Cough | 3 (3.8) | | 1 (2.4) | | 0 | | 4 (2.5) | |  |
| Nasal congestion | 3 (3.8) | | 2 (4.9) | | 0 | | 5 (3.1) | |  |
| Oropharyngeal pain | 2 (2.5) | | 0 | | 1 (2.6) | | 3 (1.9) | |  |
| Sinus congestion | 1 (1.3) | | 0 | | 1 (2.6) | | 2 (1.3) | |  |
| Skin and subcutaneous tissue disorders | | 3 (3.8) | | 1 (2.4) | | 2 (5.1) | | 6 (3.8) | |
| Acne | | 2 (2.5) | | 0 | | 0 | | 2 (1.3) | |
| Pruritus | | 1 (1.3) | | 1 (2.4) | | 0 | | 2 (1.3) | |
| Rash | | 2 (2.5) | | 1 (2.4) | | 0 | | 3 (1.9) | |

Abbreviations: AE = adverse event; mCC = menthol cigarette; mTHS = Tobacco Heating System 2.2 Menthol; SA = smoking abstinence.

Terms coded using MedDRA® version 16.0.

Supplementary Table 7. Summary of Current Cigarette Brands at Screening – Safety Population

| **Brand** | **mTHS  (N=80)  n (%)** | | **mCC  (N=41)  n (%)** | | **SA  (N=39)  n (%)** | | **Product Test  (N=5)  n (%)** | | **Overall  (N=165)  n (%)** | |
| --- | --- | --- | --- | --- | --- | --- | --- | --- | --- | --- |
| Newport | 12 (15.0) | | 9 (22.0) | | 6 (15.4) | |  | | 27 (16.4) | |
| Marlboro Menthol | 7 (8.8) | | 4 (9.8) | | 2 (5.1) | | 1 (20.0) | | 14 (8.5) | |
| Newport 100’s | 8 (10.0) | | 1 (2.4) | | 2 (5.1) | |  | | 11 (6.7) | |
| 305’s Menthol 100’s | 3 (3.8) | | 1 (2.4) | | 2 (5.1) | |  | | 6 (3.6) | |
| Marlboro Menthol 100’s | 3 (3.8) | | 2 (4.9) | |  | | 1 (20.0) | | 6 (3.6) | |
| Camel Crush Menthol | 2 (2.5) | | 1 (2.4) | | 1 (2.6) | |  | | 4 (2.4) | |
| Marlboro Black Menthol 100’s | 3 (3.8) | |  | | 1 (2.6) | |  | | 4 (2.4) | |
| Other | | 33 (41.3) | | 21 (51.2) | | 19 (48.7) | | 2 (40.0) | | 75 (45.5) |

Abbreviations: mCC = menthol cigarette; mTHS = Tobacco Heating System 2.2 Menthol; SA = smoking abstinence.

Note: “Product Test” refers to all subjects who tested the THS product but were not randomized. Only brands used by at least 4 subjects are presented.
